# Detailed Analysis of Thrombus Composition and Endovascular Thrombectomy Efficiency in Ischemic Stroke Patients with Middle Cerebral Artery Occlusion Undergoing Thrombectomy

**DOI:** 10.3390/jcm14228088

**Published:** 2025-11-14

**Authors:** Seong-Joon Lee, Mai Tuyet Nguyen, Jeong Eun Seo, Woo Sang Jung, Jin Wook Choi, So Young Park, Jin Soo Lee

**Affiliations:** 1 Department of Neurology, Ajou University School of Medicine, Suwon 16499, Republic of Korea; tienphongvp@gmail.com (M.T.N.); merykids87@gmail.com (S.Y.P.); jinsoo22@gmail.com (J.S.L.); 2 Department of Biomedical Sciences, Ajou University Graduate School of Medicine, Suwon 16499, Republic of Korea; 78945612374@naver.com; 3 Department of Radiology, Ajou University School of Medicine, Suwon 16499, Republic of Korea; stepws@naver.com (W.S.J.); radjwchoi@gmail.com (J.W.C.)

**Keywords:** thrombus histology, ischemic stroke, endovascular thrombectomy, fibrin, platelet, red blood cell

## Abstract

**Introduction:** We aimed to clarify the influence of the thrombus composition on ischemic stroke endovascular thrombectomy (EVT) efficiency by utilizing various staining methods for patients that presented with occlusions of the middle cerebral artery (MCA). **Methods:** Between September 2017 and May 2021, we analyzed thrombi retrieved during endovascular thrombectomy EVT in patients with acute ischemic stroke due to middle cerebral artery (MCA) occlusion. Patients with reperfusion failure, intracranial atherosclerotic occlusions, and inadequate staining were excluded. The thrombus composition was stratified using three staining techniques—Hematoxylin and Eosin (H&E), Martius Scarlet Blue (MSB) staining, and immunohistochemistry (IHC) for red blood cells (RBCs), white blood cells (WBCs), fibrin (Fibrin II), and platelets (CD41). Associations between EVT efficiency outcomes and the thrombus composition were evaluated. **Results:** During the study period, thrombus was available for analysis in 159 patients. A total of 59 patients were included in the main analysis. Increases in the trichotomized RBS tertiles were associated with decreases in the components of various platelet/other components but not for fibrin. A modified first pass effect (mFPE) of the modified Thrombolysis in Cerebral Infarction perfusion scale (mTICI) 2b or higher was associated with larger thrombus surface area (16.0 ± 11.6 vs. 47.4 ± 62.3 mm^2^, *p* = 0.005), a higher MSB fibrin content (29.8 ± 10.7 vs. 21.3 ± 10.9%, *p* = 0.002), and IHC fibrin (28.5 ± 14.5 vs. 20.1 ± 11.4%, *p* = 0.008). There was a marginal association between the mTICI 2b mFPE and lower MSB platelet/other components (27.6 ± 20.9 vs. 34.4 ± 14.9%, *p* = 0.078). The discrepancy between MSB platelet/others and IHC platelets was greater in the mFPE (-) group, suggesting that components other than platelets may contribute to EVT resistance. A mFPE of mTICI 2c or higher was associated with greater thrombus surface area (17.8 ± 11.9 vs. 37.7 ± 55.0 mm^2^, *p* = 0.015) and MSB fibrin (32.1 ± 10.3 vs. 22.8 ± 11.0%, *p* = 0.002). There was a marginal reverse association between the mTICI 2c mFPE and MSB RBCs (33.4 ± 20.2% vs. 41.5 ± 17.3%, *p* = 0.062). There was no significant association between final near-complete reperfusion and the thrombus composition. **Conclusions:** In patients presenting with occlusions of the MCA, a higher thrombus fibrin content is associated with better EVT efficiency. Both a higher MSB platelet/other components and RBC content may have a negative influence on EVT efficiency. These results may help identify preprocedural biomarkers beyond the conventional assessment of RBCs, WBCs, and fibrin compositions, which could guide decision-making during mechanical thrombectomy.

## 1. Introduction

Mechanical thrombectomy has brought great improvements in outcomes for acute stroke patients with large vessel occlusions [1]. These advances have enabled the histopathologic analysis of thrombi, which is expected to dramatically advance novel stroke treatment modalities and improve the secondary prevention of stroke by tracking the source of the thrombus. However, the advancement of the histopathologic analysis of thrombi is slower than anticipated due to ununified methods [2] and contradicting or insignificant results observed in multiple studies [3].

Hematoxylin and Eosin (H&E) staining can be used for the classification of thrombus composition and may be associated with a gross pathology, such as a white versus red appearance [4]. In this method, red blood cells (RBCs) appear as red, fibrin/platelets as prominent pink, and leukocytes (white blood cells, WBCs) as dark blue or purple. However, it is insufficient for discriminating fibrin and platelets. Martius Scarelet Blue (MSB) staining has been used in this regard [5] and can identify thrombus components as RBCs, WBCs, fibrin, and platelet/others. Martius Yellow, a small-molecule dye selectively stains erythrocytes and early fibrin deposits. A medium-sized molecule dye, Crystal Scarlet, and larger molecule dyes, Phosphotungstic Acid and Aniline Blue, are employed in trichrome-type staining, producing fibrin red staining. Collagen and older fibrin clusters are stained blue. Detailed immunohistochemistry (IHC) staining has further elaborated the detailed thrombus compositions. Based on such analyses, thrombus components can be largely divided into RBC-rich areas that have limited complexity, consisting of RBCs that are entangled in a meshwork of thin fibrin. In contrast, platelet-rich areas are characterized by dense fibrin structures aligned with vWF and abundant amounts of leukocytes and DNA [6].

The clinical importance of thrombus characterization lies in identifying thrombi resistant to thrombolytic or thrombectomy treatments. While thrombolysis efficiency seems to be associated with thrombus composition [4], it is somewhat limited by the post hoc nature of the thrombus acquisition after thrombolysis. Endovascular thrombectomy (EVT) efficiency, on the other hand, is more likely to be directly influenced. A number of studies have shown that platelet/fibrin-rich and RBC-poor thrombi have hostile mechanical characteristics and showed better endovascular treatment outcomes for RBC-rich thrombi [7]. However, some study results are contradictory, and a lot of the studies lack a detailed analysis of the platelet–fibrin contents and other components, as they tend to be classified as a single group due to limitations in staining methods. Few studies have performed a detailed analysis of thrombus characteristics via H&E staining, MSB staining, and detailed IHC analysis simultaneously.

Accordingly, in the current study, we aimed to identify ischemic stroke thrombus characteristics through a multimodal histopathologic analysis and to reveal their association with EVT outcomes. In contrast to earlier studies that primarily relied on a single histological technique, we planned to uniquely combine MSB and IHC staining within the same thrombus samples. This integrated approach allows for precise differentiation and the quantitative comparison of fibrin-, platelet-, and red blood cell-rich regions, enabling a cross-validated and comprehensive assessment of the thrombus architecture. Also, by comparing detailed compositional data and EVT efficiency parameters, this study aimed to provide novel insights into how histological heterogeneity may influence procedural performance and treatment outcomes [7]. To minimize various clinical and anatomic-radiologic factors that may influence the thrombus composition, patients with occlusions of a single vascular bed and homogenous stroke etiology were selected for analysis.

## 2. Materials and Methods

The current study was performed in a tertiary University Hospital that serves as a Regional Emergency Medical Center for the Gyeonggi province of the Republic of Korea. Our hospital recruited acute ischemic stroke patients with large vessel occlusion for the “Translational Research On Ischemic stroke and the role of immune Cells through Acute ischemic blood and thrombus sampling (TROICA)” registry, which consists of a prospective arm and a retrospective thrombus registry. The current data are from the retrospective thrombus registry. In detail, from September 2017 to May 2021, ischemic stroke patients undergoing EVT, in which thrombus was acquired, were included in the current study. Patients with occlusions localized to the middle cerebral artery (MCA) M1 vascular bed were included. Patients with incomplete reperfusion (modified Thrombolysis in Cerebral Infarction [mTICI] < 2b) [8] and patients with intracranial atherosclerosis-related occlusions [9] were excluded, due to the possibility of incomplete thrombus removal resulting in bias and differences in reperfusion mechanisms. After histologic thrombus analysis, patients with unsatisfactory staining were excluded from the analysis.

Ethics approval was obtained from the Ajou University Hospital International Review Board (AJOUIRB-SM-2024-429; approved on 1 September 2024), and this study was performed in accordance with the ethical standards of the 1964 Declaration of Helsinki and its later amendments. The board waived the need to obtain patient consent due the study’s retrospective nature.

### 2.1. Clinical Analysis

Patients’ clinical data was obtained from our hospital stroke registry. Neuroradiologic analyses were performed by an experienced neurointerventionist (S-J.L) blinded to histologic data. General patient demographics, time metrics, baseline imaging characteristics, endovascular treatment methods and details, inpatient outcomes, and 3-month functional outcomes by modified Rankin Scale (mRS) were collected. Occlusion site was classified based on pre-EVT noninvasive computed tomography (CT) angiography. Etiology of the large vessel occlusion was interpreted by combining TOAST criteria [10] with the intracranial atherosclerotic occlusion identification method [11]. Infarct size was stratified using the Alberta stroke program early CT score (ASPECTS) [12]. Collaterals were measured by the single-phase CT angiography method [13]. Clot burden was measured by clot burden score [14]. We also recorded the total EVT procedure time, working EVT time (from guide catheter placement at the internal carotid artery to the earliest final reperfusion image; excludes time needed for management of tandem lesions or observation time for arterial recoil), initial endovascular treatment method, number of thrombectomy attempts, successful reperfusion (classified as modified Thrombolysis in Cerebral Infarction [mTICI] Score of 2B or higher or 2c or higher [15]), or modified first pass effect ([mFPE], achieving an mTICI score of 2B or higher or 2c or higher with a single thrombectomy device pass) [16]. Early neurological deterioration (END) was classified as an increase of 2 or more points on the National Institute of Health Stroke Scale (NIHSS) during the first 7 days of early admission [17]. A 3-month mRS of 0 to 2 was considered a good functional outcome.

### 2.2. Histological Analysis

After retrieval of thrombi, they were fixed in 10% neutral buffered formalin, embedded in paraffin wax, and were cut into 4-μm sections; we included all pieces of the thrombi in the specimens. H&E staining and MSB staining were performed. IHC was performed with 3,3′-diaminobenzidine (DAB) staining for fibrin (FibII) and platelets (CD41). Stained slides were scanned at a high resolution (×400) using the Axio Scan.Z1 whole slide scanner (Carl Zeiss, Bayern, Germany). The scanned images were transformed into digital images (JPEG) for analysis of thrombus composition. All digital image segmentations and quantifications were performed by an examiner (M.T.N.) who was blinded to all clinical and procedural outcomes to minimize bias. For quantification of the thrombus components, a color-based semiautomated segmentation was performed by using ImageJ software 1.54 (NIH, Bethesda, MD, USA) for semiquantitative analysis of the percentage of red blood cells (RBCs), fibrin, white blood cells (WBCs), platelets, and other components [18,19]. They were expressed as the proportion of the total thrombus area (%). For MSB-stained images, analysis of thrombus components was performed using the color deconvolution plugin to separate the differentially stained components based on their unique colors. The components were differentiated as follows: red blood cells (RBCs) were identified using the yellow channel, fibrin was assigned to the pink channel, white blood cells (WBCs) were detected through the purple/blue channel, and platelets were recognized in the gray channel. DAB-stained images were analyzed by quantifying the intensity and area of DAB-positive staining to assess protein expression levels. The cross-sectional area of the total thrombus was also measured (mm^2^) by delineation of the clot boundary on stained histological images to represent thrombus burden [20]. To assess measurement reliability, 11 randomly selected thrombus samples were re-analyzed, demonstrating good quality intra-rater agreement (intraclass correlation coefficient = 0.988). Regions of interest (ROIs) were defined to encompass the entire visible thrombus area in each section, excluding background and tissue debris. For areas with color overlap (such as fibrin–platelet mixtures), pixel classification was determined according to the dominant hue using a standardized color deconvolution algorithm implemented in ImageJ. The quantitative results derived from MSB staining were cross-validated against corresponding IHC measurements for markers showing strong correlation (r = 0.815), confirming the consistency of the two analytical approaches.

### 2.3. Statistical Analysis

Statistical analysis was performed using IBM SPSS (version 25.0 for Windows, IBM Corp., Armonk, NY, USA). Statistical significance was set at *p* < 0.05. For a descriptive analysis of association between thrombus components and clinical features and interrelation between thrombus components, the thrombi were trichotomized according to MSB RBC tertiles [4]. For analysis of EVT efficiency by mFPE and successful reperfusion, categorical comparison was performed by chi-square test, while continuous variables were analyzed by *t*-test. The association between thrombus composition and EVT efficiency parameters were further confirmed by multiple logistic regression analyses, controlling for clinically significant variables. Comparisons of continuous variables such as IHC levels of expression and total mechanical thrombectomy passes or total procedure time were performed by correlation analysis [21]. Associations between thrombus composition and clinical outcomes were analyzed.

## 3. Results

During the study period, 360 LVO patients presented to our hospital. Among them, thrombus specimens were obtained from 159 patients. Seventy-seven individuals presented with an occlusion of the MCA M1. After the exclusion of patients with intracranial atherosclerotic occlusions (N = 11), incomplete reperfusion (N = 4), and inadequate thrombus staining (N = 3), a total of 59 patients were included in the final analysis.

### 3.1. Descriptive Analysis by RBC Tertiles

**Figure 1:**
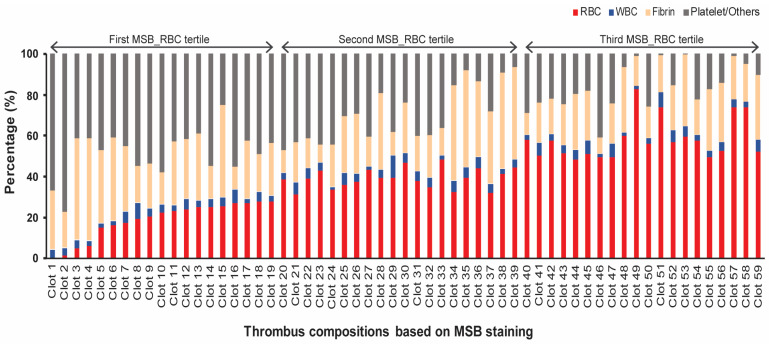
Distribution of thrombus composition based on MSB staining. Thrombus composition profiles of clots based on MSB staining are shown, stratified by RBC content tertiles. Clots are ordered from lowest to highest RBC content. The trend illustrates the inverse relationship between RBC and platelet/other fractions, while there is no such strong tendency for fibrin. MSB, Martius Scarlet Blue; RBC, red blood cell; and WBC, white blood cell.

RBC tertile-based grouping was performed to describe general histologic trends and patient characteristics, whereas specific association between each thrombus component and EVT efficiency parameter have been analyzed in subsequent sections. The distribution of four main thrombus components based on MSB staining can be seen in Figure 1. Patients were categorized into three groups based on RBC tertiles. The descriptive analyses of RBC tertiles are shown in Table 1. Increases in the trichotomized RBC tertiles were associated with decreases in the H&E fibrin/platelets/other components (72.9 ± 11.4 vs. 53.3 ± 6.8 vs. 37.8 ± 17.4%, *p* < 0.001), MSB platelet/others (48.4 ± 11.5 vs. 29.9 ± 13.7 vs. 15.8 ± 12.5%, *p* < 0.001), and IHC platelets (34.9 ± 15.8 vs. 23.4 ± 10.7 vs. 20.4 ± 10.3%, *p* = 0.002), but this was not for MSB fibrin (*p* = 0.114) or IHC fibrin (*p* = 0.085) (also refer to Figure 1). The RBC tertile was not significantly associated with stroke etiology but showed an association with large artery disease (5.3% vs. 25.0% vs. 25.0%) and tandem occlusions (0% vs. 25.0% vs. 25.0%, *p* = 0.057). It was not associated with clinical severity, risk factors, collaterals, or the clot burden. RBC tertile was not associated with EVT efficiency outcomes or functional outcomes.

The correlation analysis of MSB staining and IHC staining demonstrated a moderate correlation between MSB fibrin and IHC fibrin (R = 0.776, *p* = 0.001). MSB platelets and others showed a fair correlation with IHC platelets (*p* = 0.306, *p* = 0.019).

### 3.2. Thrombus Composition and Modified First Pass Effect

**Figure 2:**
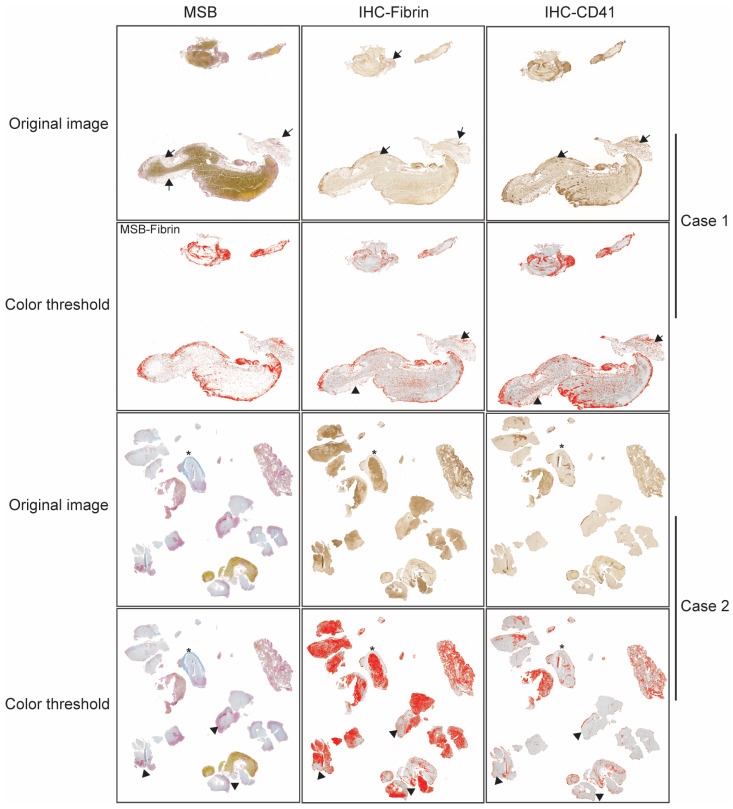
Examples of discrepancy between MSB staining and IHC for detection of fibrins, platelets, and other thrombus components from two representative cases with multiple thrombectomy trials. The first row shows original images, and second row shows corresponding images after color thresholding to highlight positive staining. Thrombus in the first case shows areas that only stain weakly in the MSB (black arrows). The corresponding area also stains weakly for IHC of fibrin and platelets. This area may correspond to connective tissue or aged fibrin. Thrombus in the second case shows areas stained as blue in the MSB (asterisk), while it does not stain for IHC of fibrin or platelets, corresponding to collagen. It also shows areas that stain pink in the MSB, corresponding to fibrin. However, they do not stain for IHC of fibrin or platelets (arrowheads). MSB, Martius Scarlet Blue; IHC, immunohistochemistry. Differences between MSB and IHC staining (notable in collagen-rich or aged fibrin regions) may indicate structural components that contribute to thrombectomy resistance.

Table 2 shows the association between the mFPE and thrombus composition. An mFPE of TICI2b or higher was associated with a larger thrombus surface area (16.0 ± 11.6 vs. 47.4 ± 62.3 mm^2^, *p* = 0.005), higher MSB fibrin contents (29.8 ± 10.7 vs. 21.3 ± 10.9%, *p* = 0.002), and IHC fibrin (28.5 ± 14.5 vs. 20.1 ± 11.4%, *p* = 0.008). There was a marginal reverse association between an mFPE of TICI2b or higher and MSB platelet/other components (27.6 ± 20.9 vs. 34.4 ± 14.9%, *p* = 0.078). This association was not observed in IHC for platelets (*p* = 0.868). The discrepancy of the average percentage between MSB platelet/others and IHC for platelets is greater in the mFPE-negative group, suggesting a higher rate of components other than platelets that do not show up well in MSB staining (Figure 2), which may contribute to EVT resistance.

To further validate these findings, we constructed a multivariable analysis adjusting for potential confounders, including age, IV thrombolysis, the clot burden score, and primary thrombectomy devices. In the model, the proportion of MSB fibrin was independently associated with achieving an mFPE of TICI2b or higher (OR = 1.09, 95% Cl [1.02–1.16], and *p* = 0.014), whereas surface area demonstrated a negative association (OR = 0.94, 95% Cl [0.90–0.99], and *p* = 0.012). After adjustment, MSB platelet/others and MSB RBC proportions showed no significant independent relationship with the mFPE of TICI2b or higher.

A more complete mFPE of TICI2c or higher was associated with larger thrombus surface area (17.8 ± 11.9 vs. 37.7 ± 55.0 mm^2^, *p* = 0.015), and greater MSB fibrin (32.1 ± 10.3 vs. 22.8 ± 11.0%, *p* = 0.002). There was a marginal reverse association between an mFPE of TICI2c or higher and MSB RBC (33.4 ± 20.2% vs. 41.5 ± 17.3%, *p* = 0.062). In the multivariable analysis, adjusting for potential confounders, including age, IV thrombolysis, clot burden scores and the primary thrombectomy device, the proportion of MSB fibrin was independently associated with the achievement an mFPE of TICI2c or higher (OR = 1.09, 95% Cl [1.02–1.16], and *p* = 0.011), while the thrombus surface area and MSB RBC contents showed no significant independent relationship with the mFPE of TICI2c or higher after adjustment.

### 3.3. Relationship Between EVT Efficiency and Thrombus Composition

A correlation analysis was performed for EVT efficiency parameters: total thrombectomy trials, the total procedure time, and the working EVT time (Table 3). Total thrombectomy trials showed a slight relationship with increases in the thrombus surface area (R = 0.310, *p* = 0.017) and decreases in MSB fibrin (R = −0.304, *p* = 0.019). The total procedure time showed a fair relationship, with decreases in MSB fibrin (R = −0.502, *p* < 0.001), IHC platelets (R = −0.361, *p* = 0.005), and IHC fibrin (R = −0.326, *p* = 0.012) and increases in the thrombus surface area (R = 0.319, *p* = 0.014); it also demonstrated a poor relation to increases in MSB RBCs (R = 0.267, *p* = 0.041) and MSB WBC (R = 0.265, *p* = 0.042). The working MT time showed a fair relationship, with decreases in MSB fibrin (R= −0.421, *p* < 0.001) and IHC fibrin (R = −0.285, *p* = 0.028), as well as a weak connection to increases in surface area (R= 0.277, *p* = 0.034)

### 3.4. Final Reperfusion Outcomes and Thrombus Composition

There was no significant association between the final near-complete reperfusion classified as mTICI2c or greater compared to the final mTICI2b reperfusion (Table 4). There was only a marginal association that suggested higher fibrin content in the near-complete reperfusion group (27.3 ± 11.1 vs. 22.1 ± 11.9%, *p* = 0.051).

### 3.5. Association Between Clinical Outcomes and Thrombus Composition

The END (N = 13) was associated with lower levels of IHC staining for platelets (CD41) (20.3 ± 9.9 vs. 27.7 ± 14.3%, *p* = 0.043). Good outcomes (N = 27) were associated with lower levels of MSB RBCs (35.1 ± 16.6 vs. 43.2 ± 19.3%, *p* = 0.048) and higher levels of IHC platelets (CD41) (29.7 ± 13.9 vs. 23.1 ± 13.1%, *p* = 0.035).

## 4. Discussions and Conclusions

The current study’s results show that, in patients who undergo EVT for occlusions of the MCA, the thrombus fibrin content measured by various methods is positively associated with EVT efficiency, especially in achieving an mFPE. This is further supported by the correlation between the fibrin content and various EVT efficiency parameters. On the other hand, a higher amount of MSB platelet/other components or RBC components may have a negative influence on EVT efficiency. The platelet/others content, measured by MSB, demonstrated a marginal negative association with mFPE 2b or higher, with a higher discrepancy in the mean MSB platelet/others fraction and the IHC fibrin fraction in the mFPE-negative group. The higher RBC content measured by MSB showed a marginal negative association with a more complete mFPE of TICI2c or higher.

A key finding of the current study is the positive association between fibrin and EVT efficiency. The positive effect of fibrin content on EVT efficiency observed in the current study seems to suggest that, in the physiological ranges of thrombosis, it consolidates the clot into one structure, making it easier to retrieve in a single pass rather than causing EVT resistance by increases in stickiness or friction [22], which can be seen in extreme laboratory conditions. Mechanistically, cross-linked fibrin structures are known to influence clot rigidity and mechanical interaction with thrombectomy devices [7,23]. Denser and more cross-linked fibrin networks may enhance the internal cohesion of the thrombus, facilitating en bloc retrieval with stent retrievers, whereas loosely organized or degraded fibrin may lead to fragmentation during extraction [24]. This may be perceived as different from previous findings, as ‘white’ thrombi with high fibrin content have been understood to be EVT resistant due to their hard organized property [25,26], supported by various ex vivo analyses of thrombus mechanical properties [7]. However, the role of fibrin itself in the formation of ‘white’ clots and thrombectomy resistance is somewhat questionable. Classic insights on the thrombus consider the thrombus of the arterial origin as platelet-rich and that of the cardiac origin, such as atrial fibrillation, as erythrocyte/fibrin-rich [27]. Furthermore, histologic analyses have revealed that fibrin is dispersed in both RBC-rich areas as a meshwork of thin fibrin and platelet-rich areas by dense fibrin structures [6]. Detailed analysis of white thrombi reveals that platelet/other components, rather than fibrin, are the main components associated with ‘white’ clots [28].

In contrast, there seems to be an association between increasing MSB platelet/other components and EVT resistance, albeit weaker. This was accompanied by a higher discrepancy in the mean MSB platelet/others fraction and IHC fibrin fraction in the mFPE-negative group. Components other than platelets may contribute to EVT resistance, as the IHC measured platelet composition did not differ. Some examples of thrombus components other than RBC, WBC, fibrin, and platelets could be collagen, which could originate from the vascular bed of the arterial thrombi [29]. A high collagen content in thrombi may result in mechanical properties resistant to EVT [30]. Another component could be tissue, for example, where there has been evidence of the presence of tumor cells or tumor-derived components within or around the clot [31]. We believe that aged and organized fibrin may also contribute to some degree in this MSB platelet/others component, but we do not have evidence to assert this opinion, as there is a moderate to strong correlation between MSB fibrin and IHC for fibrin.

The platelet/others component may also include extracellular materials such as neutrophil extracellular traps (NETs). NETs have been known to be associated with thrombolysis resistance [32], thrombus age, and longer reperfusion times [33]. We hope to address the influence of immunothrombosis on outcomes in future studies utilizing the current database.

A higher RBC content has been known to be associated with favorable EVT efficiency [26]. This phenomenon was not seen in the current study. In contrast, a tendency for an inverse relationship with first pass near-complete recanalization was seen. This is supported by other reports suggesting a fragile thrombus with increases in the RBC fraction [34,35]. This is also supported by experimental evidence that shows that the fracture resistance of fibrin-rich clots is significantly higher than red blood cell-rich clots [36]. Why the current study results do not show better thrombus removal with increases in RBC burden is debatable. The influence of RBC content may be influenced by EVT modality or advances in EVT devices and procedures. Compared to older reports using outdated devices, modern EVT devices may have better clot removability, weakening the positive influence of RBC content. A relatively uniform arterial occlusion site, the middle cerebral artery, was evaluated in the current study, which may also have influenced the results.

From a translational perspective, the current study findings may help identify preprocedural biomarkers beyond the conventional assessment of RBC, WBC, and fibrin composition that could guide decision-making during mechanical thrombectomy. Previous imaging pathology studies focused mainly on thrombus RBC content. A hyperdense MCA sign on non-contrast CT is commonly associated with RBC-rich thrombi, while the presence of a susceptibility vessel sign on MRI susceptibility-weighted imaging also correlates with RBC-rich clots due to magnetic susceptibility effects from deoxygenated hemoglobin [19]. In contrast, the absence of SVS or a non-hyperdense MCA may suggest fibrin- or platelet-rich thrombi, which are often more resistant to retrieval. However, RBC-based analyses have shown contradictory results in predicting thrombectomy efficiency. Based on the current study, future trials may need to focus on preprocedural biomarkers, allowing for the quantification of thrombus components other than RBCs, such as fibrin, platelet composition, or other components of the thrombi to predict thrombectomy efficiency. Imaging markers may also be integrated with laboratory biomarkers derived from patient blood samples, reflecting fibrin turnover, platelet activation, or thrombus aging, which may allow for the noninvasive prediction of thrombus composition and treatment response. Transcriptomic analyses of thrombus and peripheral blood may also aid in the identification of such biomarkers [37].

There are some limitations associated with the current work. First, this study is limited by a relatively modest number of cases. This is, however, also a strength of the current study. Thrombus components are known to be affected by multiple anatomic and clinical factors such as stroke etiology, tandem occlusions, intracranial atherosclerosis, etc. [24]. These factors are also known to influence EVT efficiency. Thus, the current study enrolled a targeted population of middle cerebral arterial occlusions and excluded patients with incomplete reperfusion and intracranial atherosclerotic occlusions. Second, as this study is a single-center study, it may be biased due to the selection of EVT procedures. In the current study, stent retrievers were more commonly used than aspiration catheters. Longer stent retrievers [38] or stent retrievers with open-cell designs [39] were less utilized in our hospital during the study period. Third, while this study provides further novel insights into the complexity of the fibrin–platelet–others component of the thrombus and the differential roles of fibrin and platelet/others, a more detailed histologic analysis of this complex segment is warranted in future studies. We believe that a combined detailed analysis of immunothrombosis may shed light on this complex thrombus segment. Fourth, the thrombus compositions associated with thrombectomy efficiency are not well associated with differences in outcomes. Due to the limited number of patients, a more in depth analysis should be performed to address this issue further in a larger number of patients. There is also a possibility that some baseline patient characteristics may directly influences functional outcomes and also affect thrombus composition and functional outcomes regardless of thrombectomy efficiency. Further research is needed regarding this issue.

Given emerging evidence that thrombectomy success may depend on the interaction between device type and thrombus component, future studies should also focus on thrombectomy efficiency according to devices used. In the current study, stent retrievers were more frequently used than aspiration catheters, which may partly explain the observed associations. Reporting results separately for stent retriever and aspiration techniques could clarify whether fibrin-rich or RBC-rich thrombi respond differently to each approach, but such analysis was limited by the small number of patients included.

In conclusion, in thrombi of the MCA in cases of ischemic stroke, a higher fibrin content measured by various staining methods is associated with EVT efficiency. Both increases in the MSB measured components of platelets/others and RBCs may be negatively associated with EVT efficiency.

## Figures and Tables

**Table 1:** Association between histologically classified gross thrombus appearance and clinical variables.

	1st Tertile	2nd Tertile	3rd Tertile	*p*-Value
	(N = 19)	(N = 20)	(N = 20)	
**Thrombus components**				
Surface area (mm^2^)	22.7 ± 13.5	40.4 ± 66.7	32.4 ± 45.9	0.517
H&E RBC (%)	22.8 ± 11.3	42.4 ± 6.8	57.6 ± 17.4	<0.001 *
H&E WBC (%)	4.3 ± 1.9	4.3 ± 1.9	4.6 ± 2.0	0.854
H&E fibrin/platelet/others (%)	72.9 ± 11.4	53.3 ± 6.8	37.8 ± 17.4	<0.001 *
MSB RBC (%)	18.7 ± 9.1	39.1 ± 4.9	58.6 ± 10.5	<0.001 *
MSB WBC (%)	3.8 ± 1.6	4.9 ± 3.1	4.2 ± 1.8	0.372
MSB fibrin (%)	29.1 ± 11.6	26.1 ± 14.0	21.4 ± 7.2	0.114
MSB platelet/others (%)	48.4 ± 11.5	29.9 ± 13.7	15.8 ± 12.5	<0.001 †
IHC fibrin (%)	26.7 ± 16.0	27.4 ± 14.6	18.8 ± 7.9	0.085
IHC platelet (%)	34.9 ± 15.8	23.4 ± 10.7	20.4 ± 10.3	0.002 ‡
**Clinical parameters**				
Age	73 ± 11	67 ± 15	73 ± 12	0.225
Sex, male	10 (52.6%)	15 (75.0%)	13 (65.0%)	0.345
NIHSS	18 [12–19]	15 [13–19.75]	17.5 [13.25–19.75]	0.718
HTN	15 (78.9%)	9 (45.0%)	14 (70.0%)	0.070
DM	6 (31.6%)	3 (15.0%)	3 (15.0%)	0.335
A-fib	13 (68.4%)	10 (50.0%)	12 (60.0%)	0.503
Stroke etiology				0.177
Cardioembolic	17 (89.5%)	11 (55.0%)	13 (65.0%)	
Large artery disease	1 (5.3%)	5 (25.0%)	5 (25.0%)	
Others	1 (5.3%)	4 (20.0%)	2 (10.0%)	
Tandem occlusion	0 (0.0%)	5 (25.0%)	5 (25.0%)	0.057
CT collaterals	3 [2–4]	3 [3–3.75]	2.5 [1.25–4.0]	0.541
Clot burden score	6 [6–7]	6 [5–6]	6.0 [60–6.0]	0.929
**EVT outcomes**				
Stent retrieval as primary modality	18 (94.7%)	16 (80.0%)	17 (85.0%)	0.395
Thrombectomy trials	2 [1–3]	2 [1–3]	2 [2–3]	0.917
Toral thrombectomy time, min	47 [35–60]	66.5 [35–91.5]	61.5 [45–82.25]	0.173
Working EVT time, min	32 [19–39]	31 [17.5–51.5]	37 [24.0–42.75]	0.703
mFPE (2b–3)	9 (47.4%)	11 (55.0%)	9 (45.0%)	0.804
mFPE (2c–3)	7 (36.8%)	6 (30.6%)	4 (20.0%)	0.505
**Functional outcomes**				
END	4 (21.1%)	4 (20.0%)	5 (25.0%)	0.923
Good functional outcomes	11 (61.1%)	9 (45.0%)	7 (35.0%)	0.269

* *p* < 0.001 for all comparisons, post hoc Bonferroni test. † *p* < 0.001 for comparison between 1st and 2nd and 1st and 3rd tertile; *p* = 0.002 for comparison between 2nd and 3rd tertile. ‡ *p* = 0.017 for comparison between 1st and 2nd tertile; *p* = 0.002 for comparison between 1st and 3rd tertile. H&E, Hematoxilin and Eosin; RBC, red blood cell; WBC, white blood cell; MSB, Martius Scarlet Blue; IHC, immunohistochemistry; NIHSS, National Institute of Health Stroke Scale; HTN, hypertension; DM, diabetes mellitus; A-fib, atrial fibrillation; CT, computed tomography; EVT, endovascular thrombectomy; mFPE, modified first pass effect; and END, early neurological deterioration.

**Table 2:** Association between modified first pass effect and thrombus composition.

For mFPE of mTICI 2b or Higher			
	mFPE (+)	mFPE (−)	*p* Value
	N = 29	N = 30	
Surface area (mm^2^)	16.0 ± 11.6	47.4 ± 62.3	0.005
H&E RBC (%)	40.8 ± 20.6	41.7 ± 17.5	0.431
H&E WBC (%)	4.2 ± 1.5	4.6 ± 2.3	0.216
H&E fibrin/platelet/others (%)	55.0 ± 20.3	53.7 ± 18.1	0.400
MSB RBC (%)	38.5 ± 19.7	39.8 ± 17.1%	0.399
MSB WBC (%)	4.1 ± 1.3	4.5 ± 3.0	0.230
MSB fibrin (%)	29.8 ± 10.7	21.3 ± 10.9	0.002
MSB platelet/others (%)	27.6 ± 20.9	34.4 ± 14.9	0.078
IHC fibrin (%)	28.5 ± 14.5	20.1 ± 11.4	0.008
IHC platelet (%)	26.4 ± 14.6	28.7 ± 13.1	0.868
**For mFPE of mTICI 2c or higher**			
	**mFPE (+)**	**mFPE (** **−)**	***p* Value**
	**N = 17**	**N = 42**	
Surface area (mm^2^)	17.8 ± 11.9	37.7 ± 55.0	0.015
H&E RBC (%)	36.3 ± 23.2	43.2 ± 16.9	0.102
H&E WBC (%)	4.3 ± 1.6	4.4 ± 2.1	0.409
H&E fibrin/platelet/others (%)	59.4 ± 23.0	52.3 ± 17.1	0.100
MSB RBC (%)	33.4 ± 20.2	41.5 ± 17.3	0.062
MSB WBC (%)	4.0 ± 1.3	4.4 ± 2.6	0.291
MSB fibrin (%)	32.1 ± 10.3	22.8 ± 11.0	0.002
MSB platelet/others (%)	30.4 ± 23.5	31.3 ± 16.0	0.448
IHC fibrin (%)	28.5 ± 15.9	22.5 ± 12.4	0.065
IHC platelet (%)	29.7 ± 17.4	24.6 ± 11.9	0.137

mFPE, modified first pass effect; mTICI, modified Thrombolysis in Cerebral Infarct perfusion scale; H&E, Hematoxylin and Eosin; RBC, red blood cell; WBC, white blood cell; MSB, Martius Scarlet Blue; and IHC, immunohistochemistry.

**Table 3:** Correlation between EVT efficiency parameters and thrombus composition.

For Total Thrombectomy Trials	R	*p*-Value
Surface area	0.310	0.017
MSB fibrin	−0.304	0.019
MSB platelet/others	0.109	0.413
**For Total Procedure Time**		
Surface area	0.319	0.014
MSB RBC	0.267	0.041
MSB WBC	0.265	0.042
MSB fibrin	−0.502	<0.001
MSB platelet/others	0.015	0.911
IHC fibrin	−0.326	0.012
IHC platelets	−0.361	0.005
**For Working EVT Time**		
Surface area	0.277	0.034
MSB fibrin	−0.421	<0.001
IHC Fibrin	−0.285	0.028

EVT, endovascular thrombectomy; MSB, Martius Scarlet Blue; RBC, red blood cell; WBC, white blood cell; and IHC, immunohistochemistry.

**Table 4:** Association between near-complete reperfusion and thrombus composition.

	mTICI 2c–3	mTICI 2b	*p*-Value
	N = 39	N = 20	
Surface area (mm^2^)	33.1 ± 54.2	29.8 ± 31.5	0.402
H&E RBC (%)	40.0 ± 21.4	43.7 ± 13.1	0.209
H&E WBC (%)	4.4 ± 1.9	4.4 ± 2.1	0.463
H&E fibrin/platelet/others (%)	55.6 ± 21.8	52.0 ± 12.2	0.209
MSB RBC (%)	38.5 ± 20.2	40.4 ± 14.4	0.356
MSB WBC (%)	4.0 ± 1.9	4.8 ± 3.0	0.099
MSB fibrin (%)	27.3 ± 11.1	22.1 ± 11.9	0.051
MSB platelet/others (%)	30.2 ± 19.0	32.7 ± 16.9	0.312
IHC fibrin (%)	25.5 ± 13.0	21.9 ± 14.8	0.173
IHC platelet (%)	27.6 ± 14.9	23.0 ± 10.9	0.092

mTICI, modified Thrombolysis in Cerebral Infarct perfusion scale; H&E, Hematoxylin and Eosin; RBC, red blood cell; WBC, white blood cell; MSB, Martius Scarlet Blue; and IHC, immunohistochemistry.

## Data Availability

The data supporting the findings of this study are available from the corresponding authors upon request.
